# Impact of the COVID-19 pandemic on the social and educational aspects of Saudi university students’ lives

**DOI:** 10.1371/journal.pone.0250026

**Published:** 2021-04-14

**Authors:** Abdulelah A. Alghamdi

**Affiliations:** 1 Faculty of Education, Umm al-Qura University, Makkah, Saudi Arabia; 2 Deanship of Scientific Research, Umm Al-Qura University, Makkah, Saudi Arabia; Imam Abdulrahman Bin Faisal University, SAUDI ARABIA

## Abstract

The COVID-19 pandemic led to surprising and unexpected experiences for Saudi university students. Precautionary and preventive measures taken to contain this pandemic impacted the social and educational aspects of these students’ lives. All Umm Al-Qura University (UQU) students were invited to participate in an online survey on 30 impacts, both positive and negative, of the COVID-19 pandemic on their lives. Social impact theory (SIT) was applied to illustrate these impacts. The survey yielded 1,360 responses. The results showed high to moderate levels of agreement regarding students’ perceptions of the positive and negative impact of the COVID-19 pandemic on their lives, with social aspects impacted more than educational ones; and no statistically significant gender differences. Weak correlations were found between the social aspects and the educational aspects of students’ lives in relation to the impact of the pandemic, although all aspects were correlated positively. The SIT framework provided insights into how the COVID-19 pandemic impacted students’ lives.

## 1 Introduction

Since December 2019, when it was first identified in Wuhan, the capital of China’s Hubei province, coronavirus disease 2019 (COVID-19) has spread globally, resulting in the continuing 2019–20 coronavirus pandemic [1]. Saudi Arabia confirmed its first COVID-19 case on March 2, 2020 [2] and has since has taken many drastic steps to contain the outbreak, including imposing a 24-hour curfew and closing schools and universities [3, 4]. The 24-hour curfew went into effect almost immediately in many cities, including Makkah (commonly known in the western world as Mecca) which was one of the first Saudi cities to be placed under a full-day curfew from April 2, 2020 ‘until further notice’ [5]; residents were only permitted throughout the curfew to leave their houses for essential needs between 6 a.m. and 3 p.m. within their residential area [3, 4]. The suspension of all universities and educational institutions to contain the COVID-19 outbreak was followed directly by the activation of online education during the suspension period [3, 6].

That these measures and other precautionary and preventive measures were put in place in a short period of time resulted in many inquiries about their expected consequences on students’ lives as students shifted to an at-home, virtual learning experience during the COVID-19 outbreak [6, 7]. This study aims to explore and identify the impact of the COVID-19 pandemic on the social and educational lives of Saudi university students during the period of outbreak which is ongoing until the date of conducting this study in Makkah city.

## 2 Background

### 2.1 COVID-19 outbreak in Saudi Arabia

Saudi Arabia’s experience of a previous coronavirus outbreak informed how it approached the COVID-19 outbreak. A World Health Organization (WHO) reported in 2012 the outbreak of a disease called Middle East Respiratory Syndrome-Corona Virus (MERS-COV), which spread throughout many countries globally [8]. The vast majority of MERS-COV cases were reported in the Arabian Peninsula, mainly in Saudi Arabia [9]. The outbreak of MERS-COV in Saudi Arabia began in a private hospital, but the illness subsequently spread to several hospitals; by 2014, about 25% of all Saudi MERS diagnoses were among healthcare workers [9].

The MERS-COV outbreak in Saudi Arabia demonstrated that the healthcare community was at the highest risk of infection, and students working in this field were impacted negatively. For example, a study by Al-Rabiaah et al. [10] involving 200 medical students from King Saud University concluded that there was a need to address medical students’ psychological wellbeing appropriately during the MERS-COV outbreak and suggested the establishment of psychological support programs for these students during an infectious disease outbreak. Stirling et al. [11] developed a program during an infectious period of the MERS-COV epidemic for Saudi students and faculty members of the College of Nursing at Princess Nourah University to support the emotional and informational needs of the students and staff who had the capacity to be conduits for the spread of disease to the broader population in the midst of an epidemic.

Taking into consideration the case of Saudi students during the MERS-COV intervention period from 2012 to 2014 [11], the COVID-19 outbreak scenario was approached differently, with precautionary and preventive measures taken to protect university students from infection. On March 2, 2020, the Saudi Arabian Ministry of Health confirmed the first case of COVID-19 in the kingdom. By January 10, 2021, there were 363,692 confirmed cases and 6,286 deaths had been reported in Saudi Arabia [2]. With increasing rates of COVID-19 infection, the Saudi Arabian government took quick and drastic steps to contain the outbreak. Among these urgent measures were a 24-hour curfew and the suspension of all universities and educational institutions with a shift to online education for all students [4, 6, 7].

The requirement to remain at home 24 hours per day and also continue the learning process in a different environment had the potential to impact university students’ interpersonal and intrapersonal lives both educationally and socially. Indeed, it was unprecedented globally in the educational sector for students in more than 130 countries to be out of school or university at the same time [3, 12], creating mixed feelings of perhaps sadness, confusion, worry, or fear about their future but also positively in times of uncertainty [13, 14].

Cao et al. [15] conducted a study to measure anxiety among students from Changzhi medical college during the period of the COVID-19 outbreak in China. This study showed that living in urban areas, family income stability, and living with parents were protective factors against anxiety. In addition, the results displayed that economic effects, delays in academic activities, and effects on daily life were positively associated with anxiety symptoms for students [15].

Sahu [16] highlighted the possible impact of the COVID-19 outbreak on the education of university students. Shifting from face-to-face classes to online classes is not an easy step for students, especially those who do not have access to laptops and internet facilities at home or those who take courses that cannot be taught online. In addition, students may be uncertain about assessment procedures for online assignments and projects, and will suffer when they do not have an internet facility to participate in the evaluation process, and this could adversely affect their grade averages. Such impacts of the COVID-19 outbreak on students’ education and mental health could also affect Saudi university students, especially given the many precautionary and preventive measures taken to contain the COVID-19 outbreak and prevent infection among students.

### 2.2 Research questions

In light of the identified impacts in the literature and the significance of an investigation into how students in Saudi Arabia were impacted by the COVID-19 pandemic, the following research question was devised:

In what ways did the COVID-19 pandemic outbreak in Saudi Arabia impact the lives of university students?

In order to answer this question, the following sub-question was developed to direct the scope of the study: *How do Saudi men and women university students perceive the positive and negative impacts of the COVID-19 pandemic outbreak on their lives in relation to a) social and emotional aspects*?*; b) spiritual and physical aspects*?*; c) societal and environmental aspects*?*; d) online study aspect*?*; and e) online education aspect*? Gender was an important consideration in the study for further research in this context.

## 3 Methodology

### 3.1 Procedure

Self-reported data were collected from random samples of university students in different colleges at UQU in Makkah. Ethics approval was obtained from UQU, via the Vice Presidency for Graduate Studies and Scientific Research (Ethics Approval Number: 4101130096). 5000 emails were sent by the information technology (IT) department of UQU to the accounts of university students (men and women) from April 5- to July 9, 2020. Each email included an invitation letter to take part in the anonymous online survey. In addition, an information sheet was included with each email and online consent was sought; the online survey could only be accessed after consent was submitted by participants through clicking on a ’button’ that indicated participants had read the consent information in the sheet attached to the email and agreed to participate. Some 1382 responses were received, with a response rate of 27.6%, of which 1,360 responses were complete and valid.

### 3.2 Measures

Measurement items were adapted from available literature. To ensure translation quality of measurements and their meaning equivalence, the original English version underwent two-way translation. The survey comprised three sections to obtain information related to demographic details and participants’ perceptions of the impact of COVID-19 on their lives. The socio-demographic questions covered age, gender, academic degree, and field of study. The second and third sections included the COVID-19 pandemic positive and negative impact scales. Although COVID-19 represented a novel virus and as such, had not been previously experienced, there had been similar pandemics in recent times that provided some historical literature to guide the current study. As such, the positive and negative impacts associated with the COVID-19 pandemic were elicited from available literature in relation to the impact of Severe Acute Respiratory Syndrome (SARS) and Middle East Respiratory Syndrome-Corona Virus (MERS-CoV), with some recent studies on the COVID-19 pandemic also providing guidance [16–20]. The scales gauge university students’ perceptions of the COVID-19 pandemic outbreak’s positive and negative impacts in relation to social and emotional aspects, spiritual and physical aspects, societal and environmental aspects, online study aspect, and online education aspect. These scales utilized a five-point Likert scale ranging from 1 (strongly disagree) to 5 (strongly agree).

The positive impact scale in the second section gauges the extent to which students perceived the positive impacts of the COVID-19 pandemic on their social and educational lives throughout the curfew period via 19 items: 1. Getting spiritual reflections, peace of mind; 2. Affirmation of the value of tolerance and forgiveness; 3. Solving family problems collectively; 4. Reorganization of priorities in life; 5. Cohesion among family members; 6. Feeling of societal destiny unity; 7. Awareness of importance of personal and public; 8. Attention to friends’ wellbeing; 9. Learning to take care of the body; 10. Appreciation of life and death; 11. Investment in environmental hygiene; 12. Passion for the sick and poor; 13. Enhanced sense of community contact; 14. New popular culture and humor; 15. Simulating online study to the reality; 16. Enhancing social interaction in online education; 17. Equality with all infected society members; 18. Financial and technical support in online education; 19. No effect of online study on family income.

The negative impact scale in the third section gauges the extent to which students perceived the negative impact of the COVID-19 pandemic on their social and educational lives throughout the curfew period via 11 items: 1. Low online education infrastructure; 2. Missing of classroom social environment; 3. Blurring of study plan options; 4. Inadequacy of online education for practical learning; 5. Unfair assessment in online study; 6. Discomfort and inactive physically; 7. Lack of physical movement space; 8. Burden of learning time on parents; 9. Unnecessary purchasing of material things; 10. Social alienation and distancing; 11. Fear of burden on others when infected.

### 3.3 Data analysis

Quantitative data from surveys were analyzed using the software package SPSS. Factor analysis (Principal Components analysis) was conducted on the scales to ensure items of each scale measured one representative factor using Kaiser-Meyer-Olkin (KMO) test and Bartlett’s Test of Sphericity (BTS). Descriptive analysis was applied to gauge the categorical variables’ frequencies and to determine the means and standard deviations of each scale. Independent-samples t-tests were used to determine differences between the scores of the social and educational aspects of students’ lives for men and women students, while Pearson correlation coefficients were used to explore the relationships among the aspects of social and educational lives of students. Parametric tests were considered appropriate as the sample was large, and the data met the requirements for parametric testing.

To ensure the validity of the survey, Saudi experts on the research subject reviewed and ensured the validity of the scales’ content and structure in the Arabic version. After obtaining consensus on the survey’s validity, a pilot study was conducted with a group of 25 university students to gain feedback. In addition, factor analysis (principal components analysis) was conducted on the survey scales to ensure that the items of each scale measured one representative factor using the Kaiser-Meyer-Olkin (KMO) test and Bartlett’s Test of Sphericity (BTS).

### 3.4 The research framework–Social Impact Theory (SIT)

This study relies on the presumption of the existence of social and educational impacts from launching a package of precautionary and preventive measures to contain the COVID-19 outbreak in the Saudi community and protect university students from infection. From a SIT perspective, there is the potential for changes in the social and educational aspects of students’ lives due to the impact of approved measures throughout the COVID-19 outbreak in Saudi Arabia.

Social impact can be defined as any influence on feelings, motives, behavior, or thoughts of individuals from receiving real, implied, or imagined presence or actions of others [21]. Taking this definition into account, SIT aims to explain the way in which impact is reciprocal by either a majority or a minority. Latané supports SIT as an avenue for analyzing social impact as a result of forces working in a social force field, suggesting that impact “by either a majority or a minority will be as a multiplicative function of the strength, immediacy, and number of its sources” [22]. *Strength* indicates the pervasive power from the social presence of impact sources, which differs according to authorities and positions of one impact source. The greater the strength of the source, the greater the social impact. *Immediacy* refers to closeness between the source sending information or taking action and the recipients of that information or action. More immediate sources deliver a larger social impact. Finally, the *number of sources* includes the number of sources that influence individually. The higher the number of sources, the greater the social impact consequently [21, 22].

The appeal of SIT arises from the generalizability of its framework, which can be applied in various contexts and be further tested with a particular case. Therefore, SIT has been applied in a wide variety of research areas within different contexts; for example, it was used to study: the impact of two social forces–social size and proximity–on the emotions of consumers and their self-presentation behaviors [23]; the impact of users’ number on the perceived credibility of user-generated content on social media [24]; the impact of social influence on individuals’ vaccination decision-making [25]; and the impact of relationship closeness or persuader immediacy, message persuasiveness, and perceived supportiveness on political attitude change [26].

In terms of communication and social event studies, the SIT framework provides a useful understanding for how individuals are influenced by their social environment [26]. At the same time, SIT can indicate how forces that operate in a social field are involved in events and how others are influenced by these forces over time [27]. In the current study, SIT was applied to understand how university students’ lives were influenced by the COVID-19 outbreak in Saudi Arabia in light of the precautionary and preventive measures taken in regard to the pandemic. According to the framework of SIT by Latané [21], the magnitude of social impact in this study is determined by the aforementioned factors (strength, immediacy, the number of sources), as follows.

Strength: The strength is derived from the increasing number of COVID-19 cases being identified in the Saudi Arabia. On May 1, 2020, there were 24,097 COVID-19 cases confirmed and 169 deaths from the virus in Saudi Arabia; by January 10,2021, the number of confirmed cases had increased to 363,692 with 6,286 deaths–the highest reported number among Arabian Gulf States [28].

Immediacy: With the first case of COVID-19 in Saudi Arabia reported on March 2, 2020, many precautionary and preventive measures were taken, including imposing a curfew and closing universities on March 8, 2020 [2, 4, 6, 7]. The immediacy is apparent from the closeness and connection of these measures to the aspects of community life, including university students’ lives to protect them and prevent the outbreak of the pandemic.

The number of sources: The COVID-19 outbreak in Saudi Arabia was contained through various sources (precautionary and preventive measures) surrounding the lives of university students. In terms of transport restrictions, a 24-hour curfew was imposed immediately in the holy cities of Makkah (where this study conducted) and Medina with movement restricted to only essential travel between 6 a.m. and 3 p.m. [4]. All universities and educational institutions, including public and private schools and technical and vocational training institutions, were closed [3, 6, 7]. In terms of daily social activities, all sports centers and gyms, as well as all amusement parks and entertainment zones in malls, were closed. In addition, social events, including funerals and weddings, were banned. Shopping malls, coffee shops, and public parks were closed with the exception of pharmacies and supermarkets [4].

Based on the SIT framework of Latané [21], Williams and Williams [29] postulated that social impact varies depending on whether the underlying motive for compliance is due to an external impression or an internal motive, such as self-perception. Therefore, in the current study, the framework of SIT is used as the lens to describe how all these precautionary and preventive measures affecting different aspects of students’ social and educational lives.

## 4 Results

### 4.1 Participant characteristics

Table 1 displays the demographic information of the sample. A total of 1,360 university students, both men and women, from UQU were involved in this study; these students were pursuing various academic degrees in various disciplines throughout the curfew period in Makkah, Saudi Arabia. A slightly higher number of women (52.8%) than men (47.25%) participated in the survey, with most participants aged between 19 and 23. Most of the participants were preparing for a bachelor’s degree (90.9%). The three most common fields of study among participants are social sciences (19.8%), medical sciences (17.8%), and Islamic studies (16.8%).

**Table 1:** Demographic information of the sample.

		Number of Participants	Percentage
Age	19–23	945	69.5
	24 and more	415	30.5
Gender	Man	642	47.2
	Woman	718	52.8
Academic degree	Bachelor’s	1236	90.9
	Diploma	34	2.5
	Master’s	57	4.2
	Doctorate	33	2.4
Field of study	Social Sciences	269	19.8
	Medical Sciences	242	17.8
	Islamic Studies	228	16.8
	Applied Sciences	127	9.3
	Education	111	8.2
	Arabic language	104	7.6
	Business Administration	86	6.3
	Computer	82	6.0
	Designs	69	5.1
	Engineering and Islamic Architecture	42	3.1

### 4.2 Perceptions of positive and negative impacts of COVID-19

In this study, students’ perceptions were measured through 30 statements represents the scale of the positive and negative impacts of COVID-19 pandemic on the social and educational aspects of students’ lives. These statements were subjected to principal components analysis (PCA) to ensure the validity of scale. The suitability of data for factor analysis was assessed prior to performing PCA. Many coefficients of .3 and above were revealed by inspection of the correlation matrix. The Kaiser-Meyer-Olkin (KMO) value was 0.85, exceeding the recommended value of 0.6, and the Bartlett’s Test of Sphericity (BTS) reached statistical significance (*p =* .000), supporting the factorability of the correlation matrix. PCA detected the presence of many components with eigenvalues exceeding 1. An inspection of the scree plot displayed a clear break after the second component. By using Catell’s (1966) scree test, it was decided to retain two components for further investigation. Parallel analysis supported the results of these two components with eigenvalues exceeding the corresponding criterion values for a randomly generated data matrix of the same size (30 items x 1,360). Reliability was assessed with Cronbach’s alpha, which showed an acceptable level of reliability for the items of the first component (Positive Impact) with 0.82 and the second component (Negative Impact) with 0.72. This information is presented in Tables 2 and 3.

**Table 2:** Total variance explained by the factor of the positive impact of COVID-19 pandemic items.

N*	Positive Impact Factor	Factor Loading	Eigenvalue	% Variance	Reliability Coefficient
			5.043	16.810	0.82
1	Getting spiritual reflections, peace of mind	.705			
2	Affirmation of the value of tolerance and forgiveness	.667			
3	Solving family problems collectively	.648			
4	Reorganization of priorities in life	.626			
5	Cohesion among family members	.560			
6	Feeling of societal destiny unity	.548			
7	Attention to friends’ wellbeing	.544			
8	Awareness of importance of personal and public	.543			
9	Learning to take care of the body	.535			
10	Appreciation of life and death	.514			
11	Passion for the sick and poor	.512			
12	Investment in environmental hygiene	.501			
13	Enhanced sense of community contact	.493			
14	Simulating online study to the reality	.399			
15	New popular culture and humor	.397			
16	Enhancing social interaction in online education	.377			
17	Equality with all infected society members	.320			
18	Financial and technical support in online education	.279			
19	No effect of online study on family income	.193			

Note. Kaiser-Meyer-Olkin Measure of Sampling Adequacy = 0.848, Bartlett’s Test of Sphericity, *p <* .*001*

* Order of items according to factor loading.

**Table 3:** Total variance explained by the factor of the negative impact of COVID-19 pandemic items.

N*	Negative Impact Factor	Factor Loading	Eigenvalue	% Variance	Reliability Coefficient
			3.117	10.391	0.72
1	Low online education infrastructure	.642			
2	Missing of classroom social environment	.630			
3	Blurring of study plan options	.589			
4	Inadequacy of online education for practical learning	.579			
5	Unfair assessment in online study	.568			
6	Discomfort and inactive physically	.536			
7	Lack of physical movement space	.512			
8	Burden of learning time on parents	.422			
9	Unnecessary purchasing of material things	.409			
10	Social alienation and distancing	.378			
11	Fear of burden on others when infected	.155			

Note. Kaiser-Meyer-Olkin Measure of Sampling Adequacy = 0.848, Bartlett’s Test of Sphericity, *p <* .*001*

* Order of items according to factor loading.

To determine whether the mean score of scales for each item could be described as low, medium, or high in the descriptive statistics for the impact of the COVID-19 pandemic on the social and educational aspects of students’ lives, the following descriptors were applied to the survey results: A mean below 2.5 represents a low level of agreement; a mean between 2.6 and 3.9 represents a moderate level of agreement; a mean above 4 represents a high level of agreement. The results of each scale are described in the following sections:

#### 4.2.1 Social and emotional aspects of students’ lives scale

The results from students’ perceptions in relation to the impact of the COVID-19 pandemic throughout the curfew period on the social and emotional aspects of their lives, as measured through the eight statements displayed in Table 4, showed a high level of agreement for the positive and negative impacts with an overall mean of 4.05 and a standard deviation of 0.50. This confirms that the COVID-19 pandemic affected the social and emotional aspects of students’ lives, and both positive and negative impacts were felt at the same time. In terms of gender differences, *t*-test revealed that variances for the two groups (men/women) were the same, but there was a statistically significant difference in the mean scores of men’s and women’s perceptions for the social and emotional aspects of students’ lives. However, the effect size was very small (Eta Squared = .04). Findings are presented in Table 4.

**Table 4:** Descriptive statistics for the impacts of COVID-19 on social and emotional aspects of students’ lives.

Positive Consequences					M	±SD
					4.09	± .*60*
Cohesion among family members					4.42	± .*82*
Attention to friends’ wellbeing					4.11	± .*87*
Passion for the sick and poor					4.05	± .*95*
Solving family problems collectively					3.98	± *1*.*02*
Enhanced sense of community contact					3.75	± *1*.*08*
Awareness of importance of personal and public					4.24	± .*85*
**Negative Impacts**					**3.93**	± **.*88***
Fear of burden on others when infected					4.35	± *1*.*00*
Social alienation and distancing					3.51	± *1*.*31*
**Result**					**4.05**	± **.*50***
	Overview Result			*t*-test for Equality of Means		
Gender	N	Mean	St. Dev.	t	df	Sig. (2-tailed)
Man	642	4.08	± .508	2.411	1358	.016
Woman	718	4.02	± .497	–		

#### 4.2.2 Spiritual and physical aspects of students’ lives scale

Findings displayed that students perceived a high impact (*M* = 3.97, *SD* = ±0.50) of the COVID-19 pandemic throughout the curfew period on the spiritual and physical aspects of their lives, as measured through the seven statements presented in Table 5. More specifically, the level of the negative impact of the COVID-19 pandemic was moderate (*M* = 2.93, *SD* = ±1.14), while the level of positive impact was high (*M* = 4.39, *SD* = ±0.59). In terms of the gender differences, the result of *t*-test showed that variances for the two groups (men/women) were not the same. However, there was not a statistically significant difference in the mean scores for men’s and women’s perceptions of the impact on the spiritual and physical aspects of their lives.

**Table 5:** Descriptive statistics for the impacts of COVID-19 on spiritual and physical aspects of students’ lives.

Positive Consequences					M	±SD
					4.39	± .*59*
Appreciation of life and death					4.79	± .*50*
Reorganization of priorities in life					4.50	± .*79*
Getting spiritual reflections, peace of mind					4.42	± .*89*
Affirmation of the value of tolerance and forgiveness					4.22	± .*89*
Learning to take care of the body					4.00	± *1*.*00*
**Negative Impacts**					**2.93**	± ***1*.*14***
Discomfort and inactive physically					3.22	± *1*.*29*
Lack of physical movement space					2.63	± *1*.*27*
**Result**					**3.97**	± **.*50***
	Overview Result	*t*-test for Equality of Means				
Gender	N	Mean	St. Dev.	t	df	Sig. (2-tailed)
Man	642	3.96	± .527	–	1294.5	.387
Woman	718	3.98	± .472	-.865		

#### 4.2.3 Societal and environmental aspects of students’ lives scale

The students’ responses indicated a high level of agreement (*M* = 3.98, *SD* = ±0.56) regarding the impact of the COVID-19 pandemic throughout the curfew period on the societal and environmental aspects of their lives, as measured through five statements presented in Table 6. However, the level of agreement for the negative impact (*M* = 3.29, *SD* = ±1.29) was not high as much as the level of agreement for the positive impact (*M* = 4.16, *SD* = ±0.63). For both genders, as shown by a *t*-test, the variances in students’ (men/women) perceptions were the same, with statistically significant difference in the mean scores of their perceptions of impact on the societal and environmental aspects of their lives. However, the effect size was very small (Eta Squared = .002).

**Table 6:** Descriptive statistics for the impacts of COVID-19 on societal and environmental aspects of students’ lives.

Positive Consequences					M	±SD
					4.16	± .*63*
Equality with all infected society members					4.00	± *1*.*15*
Feeling of societal destiny unity					4.22	± .*94*
New popular culture and humor					3.99	± *1*.*01*
Investment in environmental hygiene					4.41	± .*78*
**Negative Impacts**					**3.29**	± ***1*.*29***
Unnecessary purchasing of material things					3.29	± *1*.*29*
**Result**					**3.98**	± **.*56***
	Overview Result	*t*-test for Equality of Means				
Gender	N	Mean	St. Dev.	t	df	Sig. (2-tailed)
Man	642	3.96	± .574	-1.586	1358	.113
Woman	718	4.01	± .552	–		

#### 4.2.4 Online study aspect of students’ lives scale

Findings displayed that students’ perceptions confirmed a moderate level of agreement (*M* = 3.42, *SD* = ±0.78) for the impacts of the COVID-19 pandemic throughout the curfew period on their online study, as measured through the six statements presented in Table 7. In terms of gender differences, *t*-test results showed that the variances of both genders’ perceptions were the same with no statistically significant difference in the mean scores of men and women students regarding the impact of COVID-19 on their online study.

**Table 7:** Descriptive statistics for the impacts of COVID-19 on online study aspect of students’ lives.

Positive Consequences					M	±SD
					3.65	± .*89*
Simulating online study to the reality					3.79	± *1*.*09*
No effect of online study on family income					3.50	± *1*.*25*
**Negative Impacts**					**3.31**	± **.*89***
Blurring of study plan options					3.32	± *1*.*37*
Burden of learning time on parents					2.94	± *1*.*41*
Missing of classroom social environment					3.73	± *1*.*28*
Unfair assessment in online study					3.24	± *1*.*37*
**Result**					**3.42**	± **.*64***
	Overview Result			*t*-test for Equality of Means		
Gender	N	Mean	St. Dev.	t	df	Sig. (2-tailed)
Man	642	3.41	± .663	-.289	1358	.772
Woman	718	3.42	± .615	–		

#### 4.2.5 Online education aspect of students’ lives scale

Findings showed a moderate level of students’ perceptions agreement (M = 3.59, SD = ±0.61) regarding the impact of COVID-19 pandemic throughout the curfew period on the online education aspect of their lives, as measured through four statements presented in Table 8. For the gender differences, *t*-test findings showed that the variances for the two groups (men/women) were not the same. However, there was not a statistically significant difference in the mean scores for men’s and women’s perceptions of students regarding online education.

**Table 8:** Descriptive statistics for the impacts of COVID-19 on online education aspect of students’ lives.

Positive Consequences					M	±SD
					3.53	± .*91*
Financial and technical support in online education					3.46	± *1*.*21*
Enhancing social interaction in online education					3.59	± *1*.*13*
**Negative Impacts**					**3.66**	± **.*99***
Low online education infrastructure					3.43	± *1*.*24*
Inadequacy of online education for practical learning					3.89	± *1*.*21*
**Result**					**3.59**	± **.*61***
	Overview Result	*t*-test for Equality of Means				
Gender	N	Mean	St. Dev.	t	df	Sig. (2-tailed)
Man	642	3.61	± .638	–	1300.5	.398
Woman	718	3.58	± .577	.845		

### 4.3 Correlations among aspects of students’ lives

Findings of students’ perceptions showed that the social, emotional, spiritual, physical, societal, and environmental aspects of students’ lives were highly impacted, while the online study and online education aspects of students’ lives were moderately impacted by the COVID-19 pandemic, as presented in Table 9. Taking into consideration these results, a Person Correlation Coefficient (PCC) was conducted to find out the nature of the relationships among these aspects. The strength of correlation was interpreted according to the guidelines of Cohen [30], who suggests that a PCC value (*r)* from .10 to .29 indicates a weak correlation, an (*r*) from .30 to .49 indicates a medium correlation, and an (*r*) from .50 to 1.0 indicates a strong correlation. The results indicated that all relationships among all aspects of students’ lives were associated positively. The results showed that a medium to strong positive correlation existed among all social aspects (social, emotional, spiritual, physical, societal, and environmental) and between educational aspects (online study and education) of students’ lives. However, the correlation between the social and educational aspects was weak as presented in Table 9.

**Table 9:** Pearson correlations among aspects of students’ lives.

	1	2	3	4	5	M	±SD
1. Social and emotional aspects	-					4.05	± .*50*
2. Spiritual and physical aspects	0.49**	-				3.97	± .*50*
3. Societal and environmental aspects	0.42**	0.39**	-			3.98	± .*56*
4. Online study aspect	0.18**	0.26**	0.27	-		3.42	± .*64*
5. Online education aspect	0.20**	0.27**	0.29**	0.44**	-	3.59	± .*61*

** Correlation is significant at the 0.01 level (2-tailed).

## 5 Discussion

This study explores university students’ experiences regarding the impact of the COVID-19 pandemic on different aspects of their social and educational lives. These students were pursuing various academic degrees in various disciplines throughout the curfew period in Makkah, Saudi Arabia. This study setting is unique in that Makkah is the city with the third highest number of COVID-19 cases in Saudi Arabia (31,542 cases by September 8, 2020) [2]; it is also considered as the holiest city in Islam and is visited by Muslims from around the world at all times of the year; especially during Ramadan, the month of social celebration, communal worship, and performing umrah by pilgrims from around the world. This sense of togetherness, that is at the core of this city was missing in 2020 due to the pandemic [4, 31].

In terms of the period of curfew associated with the COVID-19 pandemic, Makkah has had the longest period of curfew within Saudi Arabia [5, 32]. Taking into consideration this context in the current study, the results showed a high to moderate level of agreement for the impact of the COVID-19 pandemic on different social and educational aspects of students’ lives. These results are presented and discussed in the next section.

### 5.1 Perceptions of positive and negative impacts of COVID-19

#### 5.1.1 Social and emotional aspects of students’ lives

Regarding the positive impacts, the findings in relation to the social and emotional aspects of students’ lives demonstrated that students perceived the COVID-19 pandemic helped them to be connected strongly with their family members. This result indicates that students enjoyed time with their families and that their family relationships were strong throughout the COVID-19 pandemic, which occurred during the holy month of Ramadan (April 23- to May 23, 2020), the month of social celebration and communal worship. Indeed, this finding does not support claims that in 2020, Muslims would be strongly discouraged from observing the rites of Ramadan under the shadow of the COVID-19 pandemic with mosques shuttered, collective prayers banned, and family reunions impossible [31].

On the other hand, the results related to the negative impacts indicate that students were more concerned about being a burden on others because of infection by COVID-19 than of being alone or disconnected from everyone due to the COVID-19 pandemic. This result corresponds to the nature of Saudi students’ families whereby elderly people are taken care of by their children and relatives as part of religious duty [33, 34]. While the elderly population in Saudi Arabia was most prone to complications from infection by COVID-19 [35], students experienced the burden of being a potential cause of infection of elderly people in their homes.

#### 5.1.2 Spiritual and physical aspects of students’ lives

Findings relating to spiritual and physical aspects of students’ lives confirmed that students perceived the high positive impact of the COVID-19 pandemic on their appreciation of life and death. This result reasonably coincides with the history of the coronavirus’s spread in the Arabian Peninsula [9, 36], and the increasing number of total deaths in Saudi Arabia, which ranked 30 globally with 3,956 deaths by September 2, 2020 [37]. Students also perceived the reorganization of priorities in life as a high positive impact of COVID-19, while lack of physical movement space was perceived as the least negative impact throughout the curfew period.

This result may highlight the role of information and communications technology (ICT) in reducing the need for physical movement as a result of ‘staying at home’ practices implemented by many governments to contain the COVID-19 pandemic spread and impact. In fact, ICT played an important role throughout the COVID-19 pandemic in reorganizing people’s priorities [38] and in allowing large groups of people to perform their work and study from home, enhancing their social connectedness and offering necessary entertainment [39, 40].

#### 5.1.3 Societal and environmental aspects of students’ lives

Students responded with a high level of agreement in relation to societal and environmental aspects of their lives, that keeping the investment in environmental hygiene was a positive impact of the COVID-19 pandemic for their environment. This result reflects steps taken by the Saudi government to increase the environmental sanitation campaign and sterilization of streets, public sites, and markets to curb the spread of COVID-19 [41]. For society, students’ perceptions indicated a high level of agreement for the feeling of societal destiny unity as a positive impact of the COVID-19 pandemic. This confirms that the pandemic acted as a catalyst for feelings of social unity and strengthening the connectedness of communities, despite the adversity this pandemic has also bought societies [42].

#### 5.1.4 Online study aspect of students’ lives

Findings demonstrated that online study was perceived as being more positively than negatively impacted by the COVID-19 pandemic although the level of these impacts was at a moderate level of agreement. Students found that online study was close to the reality of their learning environment throughout the curfew period. This can be attributed to the design of learning activities being suitable for the capabilities and expectations of students, related to level of increasing students’ engagement, and accessible to everyone [43]. On the other hand, missing the classroom social environment was one of the highest negative impacts of the COVID-19 pandemic on students’ online study throughout the curfew period.

This may highlight that addressing the missing social presence in online study for students through the available communication channels must be attended to by teachers to maintain and enhance the lost spontaneous student-to-student and student-to-teacher interactions. In addition, cognitive presence, which focuses on the ability of teachers to consider the preparedness of students to participate in the online study experience, and facilitatory presence through embodying direct instruction for the tools, resources and mentoring activities, are important for compensating for the missing social presence in online study [43].

#### 5.1.5 Online education aspect of students’ lives

Findings related to the online education showed that COVID-19, to a moderate level impacted positively and negatively on the online education aspect of students’ lives. Enhancing social interaction among students in online education was the main advantage of online education throughout the curfew period. The online education environment consists of two sets of interacting styles: the first one consists of students, instructor, and content, while the second consists of technologies, and methods of communication [44]. As social distancing is necessary due to COVID-19 pandemic outbreak, e-applications (such as Zoom) or Discussion Board (For Blackboard) become important primary or supportive tools for online education to help keep students connected and cope with being away from the classroom [45]. The findings of the current study confirm the positive role of online education in enhancing students’ social interactions.

However, a study conducted by Barnes and Noble College Insights with 432 college students across the U.S.A, showed that over half of the students were concerned regarding the lack of social interactions in the online learning environment, although only 12% were concerned about their speed of internet access [46]. This contradicts the findings of the current study, which showed a moderate level of negative impact reported by students in relation to the poor infrastructure of online education, with a positive impact for online education in enhancing their social interaction. Nevertheless, students’ perceptions in the current study revealed a high negative impact in terms of finding online education inadequate for practical learning throughout the COVID-19 pandemic outbreak. This highlights the necessity of shedding light on the role of technologies’ capabilities in terms of their methods of communication in supporting interaction among students and delivering a quality educational experience [44, 45, 47, 48].

### 5.2 Theoretical contribution

The findings of this study contribute to the extant literature for SIT’s framework about the impact of events in the social environment with the forces, that are involved in these events, and their influences on individuals engaged over the time of the event [26, 27]. Although this study did not target participants who were infected by COVID-19, the perceptions of current study participants showed a high to moderate level of agreement with the impact of the COVID-19 pandemic on the aspects of their social and educational lives. The COVID-19 pandemic in the framework of SIT in this study is the event that impacts on students’ lives. This impact was not immediate, but was mediated by other forces, namely the precautionary and preventive measures immediately put in place to contain the COVID-19 outbreak in Saudi Arabia.

As mentioned in the framework of SIT for this study, the precautionary and preventive measures included: imposing a curfew; closing all universities, educational institutions, and schools; closing all places of daily social activities, such as sport centers, amusement parks, and entertainment zones; banning all social gatherings, including funerals and weddings; and closing shopping malls and shops with the exception of pharmacies and supermarkets. All these precautionary and preventive measures affected different aspects of students’ lives as outlined in the next section.

#### 5.2.1 Impact of COVID-19 precautionary and preventive measures

Imposing a 24-hour curfew shifted the lives of students to a new experience, especially at home where they spent their whole day. The findings of this study indicate that staying at home helped students positively reorganize their priorities and that the period proved a positive time for spiritual reflections and achieving peace of mind, as well as providing the opportunity to be close to their family members. However, feelings of isolation and being away from their community members were confirmed as negative impacts, reflecting that an emotional part of students’ lives was absent during their experience of curfew. Closing all places of daily social activities as a precautionary and preventive measure for containing COVID-19 had an impact on students’ lives in relation to their being inactive physically and creating the feeling of a lack of space for movement, but not as much as getting an opportunity to learn how to take care of the body as a positive impact.

The banning of all social gatherings, including funerals and weddings, as a measure to contain COVID-19 coincided with the high level of agreement that a positive impact was investment in environmental hygiene. In addition, closing shopping malls and most shops received a high to moderate level of agreement by students in relation to unnecessary purchasing of material things, highlighting the impact of this precautionary and preventive measure on the style of students’ lives societally throughout the period of curfew.

Finally, university’s closure and the shift to online education for all students had a moderate impact on online study and education in their lives. In relation to the online study, students’ perceptions confirmed that experiences of online study simulated their learning environment at the university. However, online study was not able to replace the social environment of the classroom. Moreover, students found that while online education was adequate for supporting their social interaction, they missed the practical side of learning together.

#### 5.2.2 SIT’s framework and COVID-19 pandemic impacts

**Fig 1:**
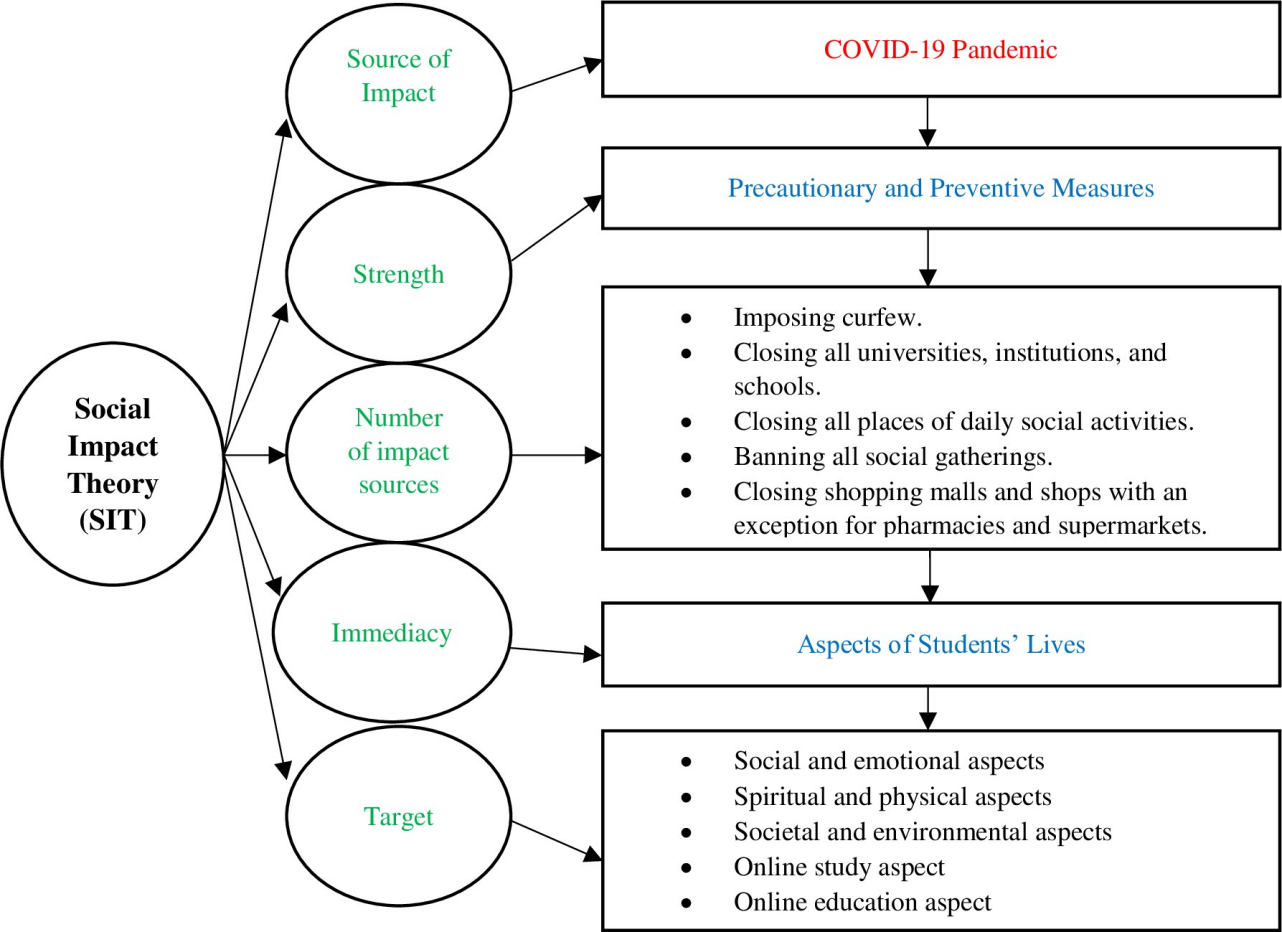
Framework of SIT for the impacts of the COVID-19 pandemic on students’ lives.

Taking into consideration students’ perceptions of the impact of COVID-19 on their lives, the SIT framework illustrates how students’ lives were affected by the COVID-19 pandemic through its three factors (strength, immediacy, and number of sources). The precautionary and preventive measures drew their *strength* as protocols that were applied to contain the COVID-19 pandemic in students’ lives but also acted as the *number of sources* impacting on the lives of the students. Furthermore, the *immediacy* of these measures related to the social roles and psychological distance of these precautionary and preventive measures in the life of Saudi students. This SIT framework for the impact of the COVID-19 pandemic on students’ lives is depicted in Fig 1.

## 6 Conclusion

Saudi students’ perceptions in this study revealed a high to moderate level of agreement with regard to the positive and negative impacts on their social and educational lives associated with the COVID-19 pandemic. However, the impact of the COVID-19 pandemic on social aspects was higher than on the educational aspects of students’ lives. Staying connected with family members, appreciating life and death, reorganizing priorities in life, practicing environmental hygiene, and feeling societal destiny unity were the prominent positive impacts that emerged. In contrast, concern over becoming a burden on others because of infection by COVID-19 was a perceived negative impact. On the other hand, aspects of online study and enhancing social interaction among students in online education were notable positive impacts on the educational lives of students throughout the period of curfew. However, missing the classroom social environment, and finding online education inadequate for practical learning were the highest reported negative impacts. The SIT framework was used to help demonstrate how the COVID-19 pandemic affected students’ lives throughout the curfew period.

## 7 Implications of this study

Although this study was conducted in Makkah, which had the third highest number of COVID-19 cases in Saudi Arabia and the longest period of curfew through the COVID-19 pandemic, the results may be similar to other international studies that suggest some positive impacts associated with the pandemic. It highlights the need for ongoing research to determine how different societies can build on the positive aspects for students that have emerged from responding to this novel virus. These positive impacts highlighted the role of information and communications technologies in reducing the negative impacts throughout the period of this pandemic. Nevertheless, these results revealed an urgent need for technologies to be developed to ensure they are in line with what users anticipate and need in their social and educational lives through crises. For example, increasing the capabilities of communications technologies to support the missing social environment in the online classroom. While it is important that the negative aspects that were highlighted are addressed, it is equally important the positive impacts that were identified are also built upon so that the benefits are not lost once society returns to pre-COVID-19 ‘normality’.

## 8 Limitations and future research

This study has a number of limitations that should be considered when interpreting the results. To begin with, the response to COVID-19 differed extensively around the world depending on location, so the results reflect the approach taken in Makkah which was even different to other cities within Saudi Arabia. Hence, similar research with different influences in relation to the place or sample could show different results. Furthermore, the results of this study were limited to differences by gender. Other factors such as year level in degrees may be worth exploring as there may have been differences between students in their preparatory year because of the need to decide on academic plans and specialties compared to those close to graduation and facing employment prospects. In addition, this study was limited to exploring the impact of the COVID-19 pandemic on the social and educational aspects of students’ lives, while further research on economic aspects could reveal different insights. Finally, interpreting how the COVID-19 pandemic impacted students’ lives within the framework of SIT was based on precautionary and preventive measures taken to contain this pandemic. However, future research could include other measures, such as following social norms and using communication technologies, which could extend the explanation of SIT’s framework for the impact of the COVID-19 pandemic on students’ lives.

## References

[1] Hui DS, I Azhar E, Madani TA, Ntoumi F, Kock R, Dar O, et al. The continuing 2019-nCoV epidemic threat of novel coronaviruses to global health—The latest 2019 novel coronavirus outbreak in Wuhan, China. International Journal of Infectious Diseases. 2020;91:264–6. 10.1016/j.ijid.2020.01.009 31953166 PMC7128332

[2] COVID 19 Dashboard: Saudi Arabia [Internet]. Kingdom of Saudi Arabia—Ministry of Health Portal 2020. Available from: https://covid19.moh.gov.sa/.

[3] Zitoun OA. COVID-19 Pandemic: Other Perspective. Saudi Arabia. International Journal of Medical Students. 2020;8(1):64–5. 10.5195/ijms.2020.50

[4] Alshammari TM, Altebainawi AF, Alenzi KA. Importance of early precautionary actions in avoiding the spread of COVID-19: Saudi Arabia as an Example. Saudi Pharmaceutical Journal. 2020;28(7):898–902. 10.1016/j.jsps.2020.05.005 32641902 PMC7242187

[5] Ministry of Interior: Curfew in All Makkah and Madinah for 24 Hours Effective from Today until Further Notice [Internet]. 2020; 2 of April. Available from: https://www.spa.gov.sa/viewfullstory.php?lang=en&newsid=2054196#:~:text=First%3A%20The%20curfew%20will%20be,of%20today%20until%20further%20notice.

[6] Alshehri YA, Mordhah N, Alsibiani S, Alsobhi S, Alnazzawi N. How the regular teaching converted to fully online teaching in saudi arabia during the coronavirus covid-19. Creative Education. 2020;11(7):985–96. 10.4236/ce.2020.117071

[7] Tanveer M, Bhaumik A, Hassan S, Ul Haq I. Covid-19 pandemic, outbreak educational sector and students online learning in Saudi Arabia. Journal of Entrepreneurship Education. 2020;23(3):1–14.

[8] Assiri A, McGeer A, Perl TM, Price CS, Al Rabeeah AA, Cummings DA, et al. Hospital outbreak of Middle East respiratory syndrome coronavirus. New England Journal of Medicine. 2013;369(5):407–16. 10.1056/NEJMoa1306742 PMC4029105 23782161

[9] Alhamlan FS, Majumder MS, Brownstein JS, Hawkins J, Al-Abdely HM, Alzahrani A, et al. Case characteristics among Middle East respiratory syndrome coronavirus outbreak and non-outbreak cases in Saudi Arabia from 2012 to 2015. BMJ open. 2017;7(1):1. 10.1136/bmjopen-2016-011865 . 28082362 PMC5253590

[10] Al-Rabiaah A, Temsah M-H, Al-Eyadhy AA, Hasan GM, Al-Zamil F, Al-Subaie S, et al. Middle East Respiratory Syndrome-Corona Virus (MERS-CoV) associated stress among medical students at a university teaching hospital in Saudi Arabia. Journal of infection and public health. 2020;13(5):687–91. 10.1016/j.jiph.2020.01.005 ; 32001194. 32001194 PMC7102651

[11] Stirling BV, Harmston J, Alsobayel H. An educational programme for nursing college staff and students during a MERS- coronavirus outbreak in Saudi Arabia. BMC nursing. 2015;14:20. 10.1186/s12912-015-0065-y . 25904821 PMC4405869

[12] Onyema EM, Eucheria NC, Obafemi FA, Sen S, Atonye FG, Sharma A, et al. Impact of Coronavirus pandemic on education. Journal of Education and Practice. 2020;11(13):108–21.

[13] Adnan M, Anwar K. Online Learning amid the COVID-19 Pandemic: Students’ Perspectives. Online Submission. 2020;2(1):45–51. 10.33902/JPSP.2020261309

[14] Garfin DR. Technology as a coping tool during the coronavirus disease 2019 (COVID‐19) pandemic: Implications and recommendations. Stress and Health. 2020;36(4):555–9. 10.1002/smi.2975 32762116 PMC7436915

[15] Cao W, Fang Z, Hou G, Han M, Xu X, Dong J, et al. The psychological impact of the COVID-19 epidemic on college students in China. Psychiatry research. 2020;287:112934. 10.1016/j.psychres.2020.112934 ; 32229390. 32229390 PMC7102633

[16] Sahu P. Closure of Universities Due to Coronavirus Disease 2019 (COVID-19): Impact on Education and Mental Health of Students and Academic Staff. 2020;12(4). 10.7759/cureus.7541 32377489 PMC7198094

[17] Chan CL. The social impact of SARS: sustainable action for the rejuvenation of society. The New Global Threat: Severe Acute Respiratory Syndrome and Its Impacts. New Jersey, United States: World Scientific; 2003. p. 123–46.

[18] Leung TTF, Lam CM, Wong H. Repositioning Risk in Social Work Education: Reflections Arising from the Threat of SARS to Social Work Students in Hong Kong during their Field Practicum. Social Work Education. 2007;26(4):389–98. 10.1080/02615470601081704 .25084357

[19] Alsubaie S, Hani Temsah M, Al-Eyadhy AA, Gossady I, Hasan GM, Al-Rabiaah A, et al. Middle East Respiratory Syndrome Coronavirus epidemic impact on healthcare workers’ risk perceptions, work and personal lives. Journal of infection in developing countries. 2019;13(10):920–6. 10.3855/jidc.11753 . 32084023

[20] Poh-Sun G, John S. A vision of the use of technology in medical education after the COVID-19 pandemic. MedEdPublish. 2020;9(1):49. 10.15694/mep.2020.000049.1 edsdoj.9b8f5410675446488c9f35323d72c0c9. PMC10697445 38058893

[21] Latané B. The psychology of social impact. American Psychologist. 1981;36(4):343–56. 10.1037/0003-066X.36.4.343

[22] Latané B, Wolf S. The social impact of majorities and minorities. Psychological Review. 1981;88(5):438–53. 0033-295X/81 /8805-0438.

[23] Argo JJ, Dahl DW, Manchanda RV. The Influence of a Mere Social Presence in a Retail Context. Journal of Consumer Research. 2005;32(2):207–12. 10.1086/432230 .18036837

[24] Mir I, Zaheer A. Verification of social impact theory claims in social media context. Journal of Internet Banking and Commerce. 2012;17(1):1–15. edselc.2-52.0–84860902619.

[25] Xia S, Liu J. A computational approach to characterizing the impact of social influence on individuals’ vaccination decision making. PloS one. 2013;8(4):e60373. 10.1371/journal.pone.0060373 . 23585835 PMC3621873

[26] Chang J-H, Zhu Y-Q, Wang S-H, Li Y-J. Would you change your mind? An empirical study of social impact theory on Facebook. Telematics & Informatics. 2018;35(1):282–92. 10.1016/j.tele.2017.11.009

[27] Lin J, Huang Y, Zhang J, Chen R. Identifying Opinion Leaders in Social Media during Brand Crises: A Case Study on Haidilao Hot Pot. Review of Integrative Business and Economics Research. 2019;8(3):24–42.

[28] (Covid-19) Disease Interactive Dashboard [Internet]. Saudi Center for Disease Prevention and Control (Weqaya). 2020 [cited 14 May]. Available from: https://covid19.cdc.gov.sa/daily-updates/.

[29] Williams KD, Williams KB. Impact of Source Strength on Two Compliance Techniques. Basic & Applied Social Psychology. 1989;10(2):149–59. 10.1207/s15324834basp1002_5 .7299143

[30] Cohen J. Statistical power analysis for the behavioral sciences. 2nd ed: Hillsdale, N.J.: L. Erlbaum Associates; 1988.

[31] AW staff. Muslims to celebrate Ramadan under the shadow of the pandemic. The Arab Weekly. 2020 22 of April.

[32] Ministry of Interior: Change to the times allowed during the curfew in all regions, except Makkah [Internet]. 2020; 26 of May. Available from: https://www.spa.gov.sa/viewfullstory.php?lang=en&newsid=2091629%232091629

[33] Sibai AM, Yamout R. Family-based old-age care in arab countries: Between tradition and modernity. Population Dynamics in Muslim Countries Springer Berlin Heidelberg; 2012. p. 63–76.

[34] Bensaid B, Grine F. Old age and elderly care: An Islamic perspective. Cultura. 2014;11(1):141–63. 10.5840/cultura20141119

[35] Al-Khudair D. Saudi Health Ministry offers COVID-19 tips for dealing with elderly. Arab News. 2020 15 of 7.

[36] Perveen S, Orfali R, Azam MSu, Aati HY, Bukhari K, Bukhari SI, et al. Coronavirus nCOVID-19: A pandemic disease and the Saudi precautions. Saudi Pharmaceutical Journal. 2020;28(7):888–97. 10.1016/j.jsps.2020.06.006 S1319016420301250. 32641901 PMC7299861

[37] COVID-19 Virus Pandemic—Worldometer [Internet]. 2020 [cited 2 of September]. Available from: https://www.worldometers.info/coronavirus/.

[38] Rolland JS. COVID‐19 Pandemic: Applying a Multisystemic Lens. Family process. 2020;59(3):922–36. 10.1111/famp.12584 32677711 PMC7404743

[39] Király O, Demetrovics Z, Potenza MN, Stein DJ, King DL, Hodgins DC, et al. Preventing problematic internet use during the COVID-19 pandemic: Consensus guidance. Comprehensive Psychiatry. 2020;100. 10.1016/j.comppsych.2020.152180 edselc.2-52.0–85084572248. 32422427 PMC7215166

[40] Alghamdi AA, Plunkett M. The Perceived Impact of Social Networking Sites and Apps on the Social Capital of Saudi Postgraduate Students: A Case Study. Future Internet. 2021;13(1):20. 10.3390/fi13010020

[41] Ministry of Media. A report On the Kingdom’s Government Efforts in the Face of the Novel Coronavirus (COVID-19). 2020 May. Report No. 2.

[42] Van Bavel JJ, Baicker K, Boggio PS, Capraro V, Cichocka A, Cikara M, et al. Using social and behavioural science to support COVID-19 pandemic response. Nature human behaviour. 2020;4(5):460–71. 10.1038/s41562-020-0884-z 32355299

[43] Rapanta C, Botturi L, Goodyear P, Guàrdia L, Koole M. Online University Teaching During and After the Covid-19 Crisis: Refocusing Teacher Presence and Learning Activity. Postdigital Science and Education. 2020. 10.1007/s42438-020-00155-y PMC7339092 40477148

[44] Seymour-Walsh AE, Bell A, Weber A, Smith T. Adapting to a new reality: COVID-19 coronavirus and online education in the health professions. Rural and Remote Health. 2020;20(2):6000. 10.22605/RRH6000 32456441

[45] Dhawan S. Online Learning: A Panacea in the Time of COVID-19 Crisis. Journal of Educational Technology Systems. 2020;49(1):5–22. 10.1177/0047239520934018

[46] Barnes & Noble Education Survey Reveals College Student Preparedness Split: Technically Ready for Online Learning, But Emotionally Unsure [Internet]. United States: Business Wire;2020; 8 of April. Available from: https://www.businesswire.com/news/home/20200408005156/en/

[47] Andel SA, de Vreede T, Spector PE, Padmanabhan B, Singh VK, de Vreede G-J. Do social features help in video-centric online learning platforms? A social presence perspective. Computers in Human Behavior. 2020;113. 10.1016/j.chb.2020.106505 S0747563220302570.

[48] Alghamdi AA, Plunkett M. Perceptions of Saudi Male and Female Postgraduate Students Regarding the Impact of Social Networking Sites and Apps on their Academic Life: A Study of Umm Al-Qura University–Makkah. International Journal of Emerging Technologies in Learning (iJET). 2018;13(05):19–40. 10.3991/ijet.v13i05.7981

